# Match-Related Acute and Residual Changes of Hip-Adduction Strength in Youth Soccer Players

**DOI:** 10.5114/jhk/204377

**Published:** 2025-09-23

**Authors:** Maziar J. Hamad, Pedro E. Alcaraz, Kristian Thorborg, Antonio Martínez Serrano, Konstantinos Spyrou

**Affiliations:** 1 UCAM Research Center for High Performance Sport, Catholic University of Murcia (UCAM), Murcia, Spain.; 2 Faculty of Sport Sciences, Catholic University of Murcia (UCAM), Murcia, Spain.; 3 Strength and Conditioning Society (SCS), Murcia, Spain.; 4 Department of Sports, Department of Orthopedic Surgery, Orthopedic Research Center Copenhagen (SORC C), Amager Hvidovre Hospital, Copenhagen University Hospital, Copenhagen, Denmark.

**Keywords:** groin, muscle strength dynamometer, youth sports, leg injury

## Abstract

The aim of this study was to investigate the changes in isometric hip-adductor strength (ADD_iso_) following official matches in youth soccer players. In this observational study, eighteen young soccer players (age: 18.03 ± 0.53 years; body mass: 69.03 ± 5.70 kg; body height: 1.75 ± 0.06 m) participated. ADD_iso_ and match external demands were measured using a hand-held dynamometer and foot-mounted inertial measurement units, respectively, during official matches (match 1, M1 and match 2, M2) at pre-match (Pre), post-match (Post), and 24 hours post (+24h). In M1, ADD_iso_ decreased significantly from Pre to Post (p = 0.003, ES = −1.14), then increased Post to +24h (p = 0.002, ES = 1.49). In M2, ADD_iso_ decreased significantly Pre to Post (p < 0.001, ES = −1.41) then increased Post to +24h (p < 0.001, ES = 2.39). Non-significant differences were found Pre to +24h for M1 (p = 1.00, ES = 0.09), while the M2 +24h time point was significantly higher than Pre (p = 0.039, ES = 0.83). Regarding external match demands, players performed significantly higher releases per min in M1 compared to M2 (p = 0.024, ES = 1.10); conversely, intense speed changes per min (accelerations and decelerations) were significantly higher in M2 (p = 0.050, ES = −0.93). ADD_iso_ decreased post-match on average between 12 and 22% in this sample of youth soccer players. The average reduction in strength was larger after M2 which may be explained by different external demands like more intense speed changes. Further research into different game intensities and their potential influence on hip-adduction strength seems relevant.

## Introduction

Groin injuries in soccer players have one of the highest injury-burdens (Bahr et al., 2018), signifying their relatively high rates of incidences and time-losses. Although a previous study (Werner et al., 2019) indicated that injury rates decreased slightly over a 15-year period, the reported injury-burden remained unchanged during the same period. Aside from the time lost from injuries, hip and groin pain are a common problem among soccer players and can lead to persisting pain along with reduced function and performance (Thorborg et al., 2017b). While traditional time-loss outcomes may only encompass one-third of cases (Harøy et al., 2017), monitoring of the presence of hip and groin pain in this population may further help capture the severity of reductions in sport-related groin function (Wollin et al., 2018b).

In soccer players, the hip-adductor muscle group is especially loaded during passing and shooting (Watanabe et al., 2020), and to even greater magnitude when stabilizing and decelerating the pelvis during change of direction actions (Dupré et al., 2021). Similarly, the common mechanism of injury seen among these high intensity actions seems to be a rapid activation of the hip-adductor muscles during lengthening contractions (Serner et al., 2019). Hip-adductor strength, as measured during isometric adduction tasks, is proposed as a modifiable injury-risk factor (Whittaker et al., 2015), and interventions which improved hip-adductor strength in male soccer players showed reductions in the prevalence of groin problems (Harøy et al., 2019). Additionally, low hip-adductor strength (i.e., at a preseason) has also shown significant associations with past and future groin pain and injuries (Markovic et al., 2020; Quintana-Cepedal et al., 2024) and the monitoring of hip-adductor strength within a competitive season has been suggested for tracking injury-risk and groin function (DeLang et al., 2023; Wollin et al., 2018b).

Alternatively, post-match changes in hip-adductor strength have previously been measured in different sports, populations and performance levels including recreational adults (Light, 2019), professional academy soccer (Springham et al., 2024), professional youth rugby (Roe et al., 2016), Australian Rules Football (Buchheit et al., 2017), and professional female field hockey during congested schedules (Sánchez-Migallón et al., 2022). Even though those studies highlight the post-match response in hip-adductor strength, more investigation is needed regarding how this change may vary in response to the different high-intensity demands of soccer matches. Specifically, knowing how hip-adduction strength is affected after an official competitive soccer match could provide objective data on exposure to potential groin injury risk and the subsequent neuromuscular recovery profiles of the hip-adductors, allowing strength and conditioning coaches as well as rehab specialists to best monitor and prepare their players while optimizing weekly training programmes.

While regular weekly strength assessments provide a feasible and objective monitoring tool, weekly measurements may miss the changes to groin strength which can occur within the microcycle and do not consider the acute and residual responses after matches. Furthermore, while acute fatigue has been shown to negatively affect modifiable risk factors of lower limb injuries of the ankle, knee, quadriceps, and hamstrings (Verschueren et al., 2020), one can hypothesize similar effects to occur to the groin region in response to acute fatigue. Additionally, in the previously mentioned systematic review (Verschueren et al., 2020), the fatigue protocols that were used in the studies were either running-based tasks (i.e., treadmill running or a soccer shuttle test to fatigue) or local fatigue protocols with closed motor tasks (i.e., repeated hip flexions). Thus, these may have lacked the ecological validity of variable and unpredictable match environments which contain dynamic demands (technical, physical, tactical, and psychological), and opponent-interactions (Bortnik et al., 2024). When considering these factors, it is important for performance and rehabilitation practitioners to understand the responses of hip-adductor strength to official soccer matches.

To the authors’ knowledge, more data are needed regarding the acute and residual post-match changes and recovery profiles of hip-adductor strength in soccer populations after official competitive matches. Considering the previously mentioned, the primary aim of this study was to assess the match-related acute and residual changes to a modifiable groin injury-risk factor, measured by hip-adductor strength. The secondary aim of this study was to investigate match external demands using foot-mounted inertial measurement units. The hypothesis of this observational study was that hip-adductor strength would decrease significantly following soccer matches and would remain diminished 24 hours post-match.

## Methods

### Participants

Eighteen healthy youth soccer players (age: 18.03 ± 0.53 years; body mass: 69.03 ± 5.70 kg; body height: 1.75 ± 0.06 m) were recruited to participate in this study. To qualify for inclusion in this study, participants had to be healthy without injury and be able to participate in the official league matches (excluding goalkeepers). Ten players participated in ≥ 60 min in match 1 (M_1_) (18.20 ± 0.25 years, body mass: 70.14 ± 6.37; body height: 1.76 ± 0.06) and nine players in M_2_ (18.18 ± 0.26 years; body mass: 69.40 ± 6.82; body height: 1.75 ± 0.06) as one player was withdrawn due to a first-half injury. Additionally, at the 24-hour time point, three players were unavailable to come into the lab for testing due to personal reasons, therefore, sample sizes at Pre, Post, and 24-h post-match time points were N = 10, N = 10, N = 7 for M_1_, and N = 9, N = 9, N = 6, for M_2_ respectively. All participants were informed about the procedures, details, and risks of the study and all provided their written consent. Legal guardians signed informed consent for any participant who was a minor. The study was completed according to the Declaration of Helsinki (2013) and approved by the institutional ethics committee of the Catholic University of Murcia (UCAM), Murcia, Spain (protocol code: CE042410, approval date: 26 April 2024).

### Measures

#### Isometric Hip-Adduction Strength (ADD_iso_)

To assess isometric hip-adductor strength (ADD_iso_), measurements were done on a physiotherapy bench in a supine position, with the tested limb fully extended and the non-tested limb flexed at the knee, while participants secured themselves by grabbing the sides of the bench. Before each maximal test, players performed two warm-up adduction repetitions per limb at 50% and 75% of their perceived maximal intensity for 5 s. The instructions given to participants were to “apply force into the dynamometer as hard as possible”. Following the warm-up, participants performed two maximal unilateral, 5-s hip-adduction efforts per limb while strength was measured using a hand-held dynamometer (HHD) (KFORCE Muscle Controller, Kinvent Biomecanique, Montpellier, France) by the same tester each time and according to previously validated methods (Thorborg et al., 2010). The measurement of hip-adduction strength using the HHD is both reliable and validated in soccer players when compared to fixed, laboratory-setting dynamometers (Mentiplay et al., 2015). During each trial, players were verbally encouraged, and 10 s of rest was provided after each repetition, and 30 s of recovery was given before switching limbs. The HHD provided peak force data in kg during each trial, which were then converted to relative torque (Nm/kg) using each participant’s limb length (m) and body mass (kg). Limb length was measured from the anterior superior iliac spine to 5 cm superior to the lateral malleolus (adjacent to the point of application of the HHD). The reliability of the HHD strength test between repetitions and between days was assessed for each limb (dominant and non-dominant) one week before the first match on two separate familiarization days. Inter-repetition reliability was established between repetitions for 16 players on the same day; the intraclass correlation coefficient (ICC) results corresponding to ICC_(3,1)_ were: 0.91 (dominant) and 0.91 (non-dominant); within-subject coefficients of variation (CV) were: 3.91 ± 2.85% and 4.99 ± 2.47%, respectively. Inter-day reliability was established between days for 10 players on two separate days; the intraclass correlation results corresponding to ICC_(3,1)_ were: 0.79 (dominant) and 0.84 (non-dominant); within-subject CVs were: 8.74 ± 7.06% and 6.90 ± 6.32%, respectively.

#### External Match Demands

The external locomotor demands during each match were measured using foot-mounted inertial measurement units (IMU) (Playermaker^TM^). The IMUs contained a 16-g, 3-axis gyroscope, and a 3-axis accelerometer to quantify the angular velocity and acceleration of each foot, respectively. Each IMU was encased in a silicone sleeve that was worn on each soccer cleat during the match. The Playermaker^TM^ was turned on 10 min before the match and did not require any prior calibration. The Playermaker^TM^ has shown good to excellent between-unit reliability and good validity for measuring locomotor characteristics (Myhill et al., 2023). Match demands included participation time (min), total number of releases (passes, shots), number of releases 0–15 m/s (zones 1–3), number of releases >15 m/s (zones 4–6), work rate (m/min), high-intensity distances >4 m/s (HID) (m), sprint distances >5.5 m/s (SpD) (m), sprint count (#), and intense speed changes (i.e., the number of acceleration and deceleration actions) >2.6 m/s^2^ (#). Warm-ups were not included in the data acquisition. All absolute external demands were then normalized to playing time (/min).

#### Groin Pain

To assess sports-related hip and groin function, immediately after completion of the isometric tests, participants were asked to rate their pain for each limb during the hip-adduction efforts according to the Copenhagen five-second squeeze protocol (Thorborg et al., 2017a). Participants were asked to rate their groin pain on a numerical pain rating scale, from 0 to 10, such that ‘0’ represented no pain, and ‘10’ represented maximal pain.

### Design and Procedures

**Figure 1:**
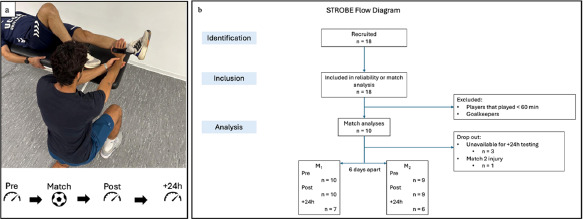
. Study design. 1a, Testing setup and timepoints of isometric adductor strength; 1b, Flow diagram of recruitment, inclusion and analysis

The present study followed the STROBE statement and its accompanying checklist to guide the reporting of an observational study (von Elm et al., 2007). The study had a prospective, repeated-measures design aiming to investigate the changes in hip-adductor strength involving two official league matches separated by one match-week (i.e., six days) during the 2023/2024 competitive season. Match 1 (M_1_) took place on May 19, 2024, and match 2 (M_2_) on May 25, 2024. Hip-adductor isometric strength, groin pain, and external match demands during matches were measured during the study. Only outfield players who participated in ≥ 60 min during the match were included in the subsequent analyses. To familiarize the participants with all the procedures, two familiarization sessions (two days apart) were conducted one week before the first match. For match-testing, participants were evaluated 30 min pre-match (Pre), within 10 min of termination of the match or being substituted out (Post), and 24-h after the match, ± 1h (+24h) (Figure 1)

### Statistical Analysis

Normality was assessed using a Shapiro-Wilk test. All variables were normally distributed and their descriptive data were presented as mean ± SD except pain which was non-normally distributed and was presented as median and interquartile range. To assess if Pre-match ADD_iso_ was different between the two matches, a Student’s *t*-test was carried out between M_1_ and M_2_. External demands between the two matches appeared to have unequal variances (Levene’s test) and were therefore analysed by a Welch’s *t* test. The peak torque from the best trial for each limb was used for analyses and the dependent variable was the maximum adduction torque for each limb. A linear mixed model was used to assess the time course changes in ADD_iso_ at Pre, Post, and +24h. The model terms were tested with a Satterthwaite test method, with Limb (dominant, non-dominant) and Time (Pre, Post, and +24h) used as fixed effects and each participant as a random effect grouping factor. When any statistically significant main or interaction effects were found, a post hoc analysis was performed with Bonferroni adjustment. Effect sizes and their 95% confidence intervals were reported as Hedges’ g_av_ following the guidelines by Lakens (2013). The level of significance was set at ≤0.05. Statistical analysis was done in JASP, JASP Team (2024), JASP (Version 0.18.3) [Computer software] (JASP Team, 2024).

## Results

No significant differences were observed in peak relative torque (Nm/kg) between the two matches at time point Pre (t (36) = 0.731, *p* = 0.469, ES = 0.23 [−0.41, 0.87]).

### Match 1

**Figure 2:**
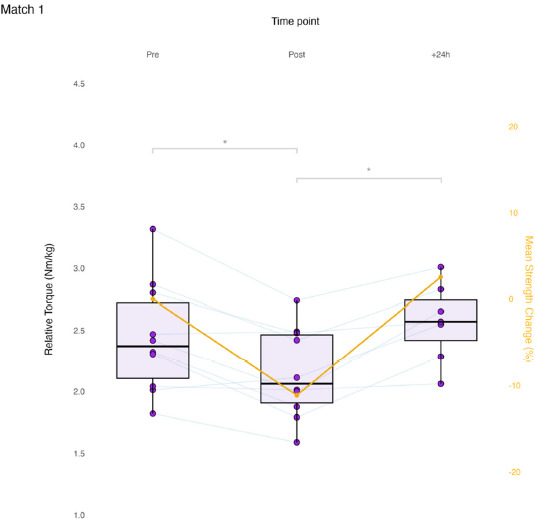
Match 1 time course changes in maximum isometric hip-adduction strength. Each data point is the limb-averaged relative torque (boxplots, left axis). Average percent changes are presented relative to pre-match (line plot, right axis). Pre and Post, N = 10; +24h, N = 7; * p < 0.05

Results of the linear mixed model for ADD_iso_ showed a significant effect of Time for M_1_ (F (2, 39.42) = 9.212, *p* < 0.001). At time point Post, ADD_iso_ decreased significantly from Pre in M_1_ (mean diff. −0.29 Nm/kg, *p* = 0.003, ES = −1.14 [ −1.91, −0.34]). From Post to +24h, ADD_iso_ increased significantly in M_1_ (0.34 Nm/kg, *p* = 0.002, ES = 1.49, [0.39, 2.55]). Between Pre and +24h, no significant differences were present in M_1_ (0.05 Nm/kg, *p* = 1.00, ES = 0.09, [−0.62, 0.80]). No significant effect was found in the change in ADD_iso_ by the Limb factor (i.e., dominant and non-dominant) for M_1_ (F(1, 38.97) = 0.002, *p* = 0.967). No significant effect of a Time * Limb interaction was found in M_1_ (F(2, 38.97) = 0.097, *p* = 0.908). Match 1 time course changes are presented in Figure 2.

### Match 2

**Figure 3:**
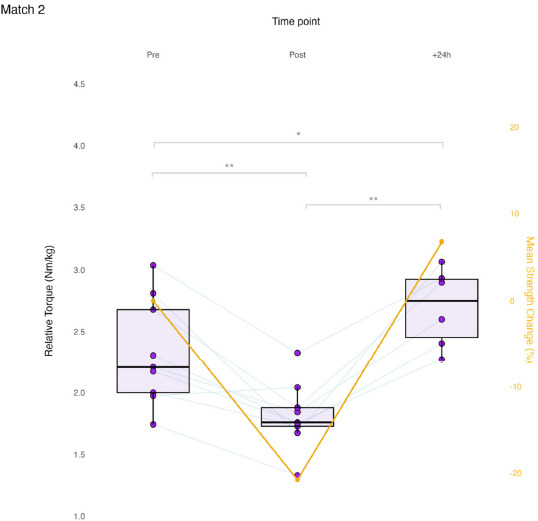
Match 2 time course changes in maximum isometric hip-adduction strength. Each data point is the limb-averaged relative torque (boxplots, left axis). Average percent changes are presented relative to pre-match (line plot, right axis). Pre and Post, N = 9; +24h, N = 6; * p < 0.05; ** p < 0.001

Results of the linear mixed model for ADD_iso_ showed a significant effect of Time for M_2_ (F (2, 34.59) = 31.694, *p* < 0.001). At time point Post, ADD_iso_ decreased significantly from Pre in M_2_ (−0.51 Nm/kg, *p* < 0.001, ES = −1.41, [−2.31, −0.48]). From Post to +24h, ADD_iso_ increased significantly in M_2_ (0.78 Nm/kg, *p* < 0.001, ES = 2.39, [0.76, 4.00]). Match 2’s +24h time point was significantly higher than Pre (0.28 Nm/kg, *p* = 0.039, ES = 0.83, [−0.10, 1.70]). No significant effect was found in the change in ADD_iso_ by the Limb factor (i.e., dominant and non-dominant) for M_2_ (F(1, 33.90) = 1.494, *p* = 0.230). No significant effect of a Time * Limb interaction was found for M_2_ (F(2, 33.90) = 0.442, *p* = 0.646). Match 2 time course changes are presented in Figure 3.

The results of the analysis of external match demands are listed in Table 1. M_1_ presented significantly higher values in releases per min (*p* = 0.024, ES = 1.10 [0.09, 2.08]), and releases per minute within zones 4–6 (*p* = 0.045, ES = 0.96 [−0.03, 1.92]). M_2_ showed significantly higher values in intense speed changes per min (*p* = 0.050, ES = −0.93 [−1.87, 0.05]). Descriptive results for the maximum pain during the hip-adduction tests are presented in Table 2.

**Table 1:** External demands normalized to playing time.

	Match 1 (N = 10)	Match 2 (N = 9)	% Difference (*M*_1_ − *M*_2_)	ES [95%CI]	*p*
Player participation time (min)	87.00 ± 10.54	86.44 ± 20.28	1.11 ± 16.30	−0.17 [−0.74,0.54]	0.714
Releases per min (#/min)	0.45 ± 0.21	0.27 ± 0.09	36.00 ± 56.46	1.10 [0.09, 2.08]	0.024^*^
Zones 1–3 releases per min (#/min)	0.24 ± 0.12	0.15 ± 0.07	8.26 ± 43.51	0.91 [−0.07, 1.85]	0.054
Zones 4–6 releases per min (#/min)	0.21 ± 0.12	0.12 ± 0.05	33.94 ± 67.00	0.96 [−0.03, 1.92]	0.045^*^
Work rate (m/min)	98.55 ± 6.67	97.69 ± 6.54	0.89 ± 11.27	0.12 [−0.78, 1.02]	0.780
HID covered per min (m/min)	16.90 ± 6.00	19.25 ± 2.62	−5.90 ± 24.19	−0.49 [−1.40, 0.44]	0.282
SpD covered per min (m/min)	4.28 ± 2.01	4.49 ± 1.29	3.67 ± 53.07	−0.12 [−1.02, 0.78]	0.783
Sprints per min (#/min)	0.43 ± 0.18	0.49 ± 0.11	−3.29 ± 39.43	−0.37 [−1.28, 0.55]	0.409
Intense speed changes per min (#/min)	0.76 ± 0.33	1.02 ± 0.19	−23.01 ± 42.25	−0.93 [−1.87, 0.05]	0.050^*^

Summary data are presented as mean ± SD; * Significant at ≤ 0.05; Zones 1–3, (0–15 m/s); Zones 4–6 (>15 m/s); HID, high intensity distance (>4 m/s); SpD, sprint distance (>5.5 m/s); Intense speed changes are defined as accel/decel actions >2.6 m/s^2^

**Table 2:** Frequency table of maximum pain scores reported during the isometric adduction test for players playing ≥ 60 min.

	Match 1		Match 2	
Pain scores *	Pre (N = 10)	Post (N = 10)	Pre (N = 10)	Post (N = 9)
0–2	0	1	1	0
3–5	8	5	8	2
6–10	2	4	1	7
Median (IQR)	5.0 (3.2–5.0)	5.00 (3.2–6.0)	5.00 (5.0–5.0)	6.00 (6.0–7.0)

* Pain scores are based on a numerical pain rating scale from 0 to 10, with ‘0’ being “no pain” and ‘10’ being “maximum pain”

## Discussion

The aim of this study was to provide an exploratory analysis of the changes in hip-adductor strength before and after official soccer matches in youth players. The main finding of this study was that in this group of youth soccer players, ADD_iso_ consistently decreased after a match and then recovered by +24h. The average post-match reduction in strength was 12% in M_1_ and 22% in M_2_. Groin strength was recovered by +24h in both matches and in one match, +24h strength was higher compared to pre-match (baseline). The results from this study provide objective data regarding the match-related neuromuscular responses in hip-adduction strength; this study provides practitioners with relevant information on how to plan training and return to play protocols.

Regarding pre- to post-match profiles, our results are similar to those seen in a previous study conducted with recreational male soccer players, where a 17.7% reduction in ADD_iso_ immediately following the match was found (Light, 2019). Interestingly, in that study, reductions were apparent by 45 min as a roughly 10% reduction was reported at halftime (Light, 2019). In another study of professional academy soccer players (Springham et al., 2024), the authors observed statistically significant and *moderate* (ES = 1.13) reductions post-match relative to pre. While this reduction was similar to that of our study (ES = 1.14 and 1.41, Post M_1_ and M_2_), it is important to note the study by Springham et al. (2024) included professional academy (category 1) players and measured ADD_iso_ in a shorter lever position (knee), while we measured it in a longer lever (ankle); the long lever position may have higher sensitivity to detect strength changes (Light and Thorborg, 2016). Similarly, during fixture congestion, such as in a tournament setting, roughly 72% of a youth squad experienced meaningful ADD_iso_ reductions (>15%) (Wollin et al., 2018a). As previously mentioned, when neuromuscular responses were measured after different tactically-periodized training sessions (i.e., strength, endurance, and speed) in a group of elite French soccer players (17 ± 2 years), Buchheit and colleagues (2018) observed decreases in groin squeeze of −12%, −7%, and −7% in strength, endurance, and speed, respectively. Our results interpreted along with theirs may reinforce the differences in groin-strength demands and between training and matches and can also describe which types of training sessions more similarly expose the groin region to match-like demands. It is hoped that this can help coaches and rehabilitation practitioners prescribe relevant training interventions to fit their intended outcomes within clinical and performance settings.

Regarding the residual changes (≥ 24 hours), in our study by +24h, ADD_iso_ was not significantly different from pre-match in M_1_ and in M_2_ there appeared a slight increase in strength compared to pre-match levels (2.70 vs. 2.33 Nm/kg) signifying a possible supercompensation effect. Similarly to our study, a small supercompensation effect in ADD_iso_ has also been seen before in academy rugby union players at 48-hours post-match (Roe et al., 2016). In the study by Springham and colleagues (2024), a +24h measurement was not performed, but at MD+2 and MD+3 ADD_iso_ continued to show *small* reductions. In other sport populations, female field hockey players during a tournament saw post-match decreases in isometric adduction of 10.6% after the first match and a 17.5% decrease relative to pre-match 1 after the second match on the following day (Sánchez- Migallón et al., 2022). In a sample of Australian Rules Football, Buchheit and colleagues (2017) saw a 17.4% reduction in isometric adduction strength on the following day after a match compared to baseline. The observations that groin strength was recovered by 24 h in both matches may have been due to the task-specific demands of the isometric test at shorter muscle lengths, as perhaps isometric strength may have been a less sensitive measure (to detect fatigue) at 24 h post-match, when compared to either isometric strength at longer muscle lengths or eccentric strength (Thorborg et al., 2014). These findings suggest that the recovery response may vary among different populations and that by 24 h, strength may be reduced, recovered or in some cases increased beyond baseline values. Therefore, determining the adequate timing and dose-response relationship of groin strength to training stimuli could be insightful to reduce injury risk, additionally, a standardized ADD_iso_ testing protocol is important to allow for more direct comparisons among studies.

M_1_ seemed to be significantly higher on average in technical actions such as the number of releases per min (ES = 1.10), and the number of releases within zones 4–6 (>15 m/s) (ES = 0.96). While not statistically significant at an alpha set at the 0.05 level, M_1_ also seemed higher in the work rate (m/min), and releases per min within zones 1–3 (0–15 m/s) (ES: 0.12 and 0.91, respectively). M_2_ presented a significantly greater number of intense speed changes per min (ES = 0.93). While not reaching statistical significance, other high-intensity actions such as HID covered per min, SpD per min, and the number of sprints per min were all greater in M_2_ (ES: −0.49, −0.12, and −0.37, respectively). These results seem to convey that soccer matches may not be created equally with respect to their demands and that soccer matches may result in a wide range of decreases in groin strength. While caution should be taken in interpreting these results due to small sample sizes, this may further highlight that matches predominantly higher in intense speed changes (accelerations and decelerations) may result in greater reductions in groin strength than matches more dominated by greater distances and technical demands. Our estimation is in line with similar research that showed that the greatest reduction in groin strength was seen in the sessions that favoured more concentrated playing areas (higher player to playing area ratio) (Buchheit et al., 2018), possibly since concentrated playing areas have shown to correlate to higher numbers of changes of direction actions (de Dios-Álvarez er al., 2025). This is further supported by biomechanical data showing that groin muscle forces were greater during cutting maneuvers than inside passing tasks (Dupré et al., 2021).

With respect to hip and groin pain, cases seemed common in this sample of youth soccer players, with median pre-match pain being 5 and at least 90% of match-day players reporting pain ≥ 3, on a numerical pain scale from 0 to 10. Median post-match pain in M_2_ seemed higher with 70% of the players reporting pain between 6 and 10. This may partially explain why groin strength decreased more in M_2_ as it has been shown that pain may have an inhibitory effect on groin strength (DeLang et al., 2023; Thorborg et al., 2014), and that subsequent reductions in pain are seen in conjunction with a recovery in groin strength (DeLang et al., 2023). A high prevalence of groin pain has previously been reported in soccer populations, ranging between 49% and 67% in adult soccer players (Hanna et al., 2010; Thorborg et al., 2017b). In this sample of youth soccer players, nearly all of the match-day players (>90%) reported groin pain above 3. According to the guidelines outlined by Thorborg et al. (2017a), pain scores between 3 and 5 indicate that players should receive attention and be reviewed by a health professional, while scores between 6 and 10 indicate severe disability and clinical workup. In our sample, the frequencies of those reporting pain of 3–5 and 6–10 pre-match was 80% and between 10 and 20%, respectively.

It is acknowledged that the present study has some limitations inherent to the nature of conducting research during official soccer matches and sports settings. Our study was limited by its naturally small sample size. Having a maximum of only ten players available to participate in each match may mean excessive variability in the results and it limits the generalizability of the findings to a broader population. Additionally, having a small sample size limits the ability to subgroup the data to determine possible moderating factors as dividing an already small sample size increases the risk of obtaining false positives. Additionally, the limited number of observed matches (i.e., two) may not have been able to capture the entire range of game demands and physiological effects; yet despite this, this has also been a strength of the current study as it reflects the more relevant demands from official games instead of closed tasks or drills. Another limitation of this study is that since an isometric test was used, the reduction and recovery profile of groin strength is limited to this mode of testing and may not be generalizable to another contraction mode like eccentric strength. Finally, another limitation was that only peak hip-adduction force was measured without considering a possibly more sensitive metric such as the rate of force development for the detection of fatigue. Further research should consider including these metrics in the analysis.

## Conclusions

In this sample of youth soccer players, isometric hip-adductor strength decreased on average between 12 and 22% after official soccer matches. By 24 h post-match, groin strength seemed to have recovered and at times even experienced a supercompensation in comparison to pre-match values. To determine the factors that impose the greatest load on the groin region and that may increase injury-risk, future research should consider the demands on hip-adductor strength after games and training sessions of varying intensities and contexts. The findings of the present study may help strength and conditioning coaches and rehabilitation practitioners understand and identify the hip-adductor strength demands of different soccer matches. Our data support monitoring decisions and frameworks within the competitive microcycle. Based on the present results and discussion, practitioners may wish to limit exposure to sessions that have high intensity cutting or speed changes during congested competition periods or in those in early-stage hip-adductor rehab.
